# Long-Term Outcomes of Pediatric Kidney Transplants From DCD and DBD Donors: A Comparative OPTN Study

**DOI:** 10.3389/ti.2025.14706

**Published:** 2025-10-09

**Authors:** Alicia Paessler, Joe Brierley, Marion Siebelink, Ioannis Loukopoulos, Nicos Kessaris, Jelena Stojanovic

**Affiliations:** ^1^ Great Ormond Street Hospital for Children NHS Foundation Trust, London, United Kingdom; ^2^ Transplant Centre, University Medical Center Groningen, Groningen, Netherlands; ^3^ Guys and St Thomas’ NHS Foundation Trust, London, United Kingdom; ^4^ University College London (UCL) Institute of Child Health, London, United Kingdom

**Keywords:** registry, kidney transplant, DCD, pediatric, DBD donor

## Abstract

We compared the long-term outcomes of pediatric kidney transplants from DCD and DBD donors over a 33-year period in the USA. Data were retrieved and analysed on kidney transplants from deceased donors in paediatric recipients in 1994–2020 from the OPTN. Data were compared between those receiving kidney transplants from DBD and DCD donors. There were 11,071 paediatric kidney transplants from deceased donors including 350 from DCD donors. DCD transplants were more likely to have delayed allograft function (20.1% vs. 11.9%, p < 0.01). However, there was no significant difference in allograft or patient survival between transplants from DBD and DCD donors at 10 years (56% vs. 55%, p = 0.76 and 90% vs. 91%, p = 0.89). We describe the largest cohort of pediatric DCD kidney transplant recipients in the literature. We showed that despite higher rates of delayed allograft function in DCD transplants, long-term outcomes were not significantly different. Kidney transplants from DCD donors are a viable option and should be offered to children comparable to DBD kidneys as their long-term outcomes do not differ. DCD transplantation is illegal in some countries, however, it offers an opportunity to increase the number of transplants for children; this data should be considered in ongoing policy discussions.

## Graphical Abstract

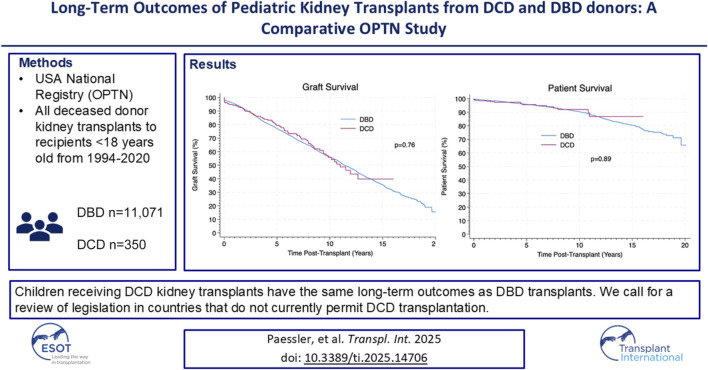

## Introduction

As modern medicine advances, there is an increasing number of children with complex conditions surviving outside infancy, who are now presenting to transplant professionals as potential transplant candidates. Over the last 20 years there has been a 54% increase in children being listed for a kidney transplant from a deceased donor in the USA [1]. Unfortunately, transplant rates have not increased to the same degree, leaving children waiting for organs longer than previously. In 2023, 29% of children on the waiting list had been waiting for longer than 2 years for their transplant [1]. Furthermore, 16 children were removed from the waiting list that year because they had died or were too sick for transplantation [1]. There is a clear need to increase the potential donor pool for paediatric kidney transplant recipients.

One of the things that has become increasingly common to increase the donor pool is using organs that have been donated after circulatory determination of death (DCD). The difference between DCD and DBD (donation after brainstem determination of death) is the criteria used to confirm the death of the donor–either circulatory or brainstem. DCD rates rapidly declined in the USA after brain death legislation was adopted in 1968, although the interest in DCD began to increase again in the 1990s as a way to meet the increasing demand for donor organs; largely led by the Maastricht team in the Netherlands [2, 3].

There is still a large variation in DCD practice across the world with some countries such as the USA, UK and the Netherlands, predominantly practicing controlled DCD - donation occurs after cardiac arrest in donors following withdrawal of life-sustaining therapy [4]. In other countries such as France and Spain uncontrolled DCD is also practiced–donation following unsuccessful resuscitation attempts in the donor [4]. However, there are also several other countries such as Germany [5], Greece [6], Hungary [7] and others, which do not allow any form of DCD [5, 8]. There is now significant global experience in the safe performance of DCD in children, with the key concept of the permanence of terminal cardiorespiratory arrest based on the concept of loss of function that can resume spontaneously, and a decision that there will be no resuscitation attempts. One of the issues with such different donation practices is that it has somewhat limited the amount of evidence produced on the long-term outcomes of DCD transplantation. DCD transplantation is widely accepted in adult practice in countries which permit it. As adults represent a much higher proportion of overall transplant activity than children, there are many studies with large datasets looking at the long-term outcomes of DCD in adults, these are unfortunately lacking in paediatrics so far. These large-scale studies have shown positive results with DCD transplants having equivocal allograft survival, despite initially higher rates of delayed allograft function (DGF) [9, 10]. However, partially due to a lack of the same positive evidence and the lack of large-scale studies in paediatrics, to this day there is some skepticism among some professionals on the long-term effects associated with DCD organs in paediatrics. There remains a concern amongst some clinicians about perceived increased risk of primary non-function, DGF and long-term allograft outcomes. The largest study to date on paediatric DCD transplants included less than 300 patients and found increased rates of DGF, with another study finding increased rates of late allograft loss [11, 12]. DCD kidneys sustain a warm ischaemic injury around the time of donation, this occurs due to the period of hypoperfusion that happens between the withdrawal of life-sustaining treatment until asystole. This is then further compounded by the period of no perfusion during the mandated stand-off time between asystole and the retrieval. This warm ischaemic injury is thought to contribute significantly to the previously reported higher rates of delayed allograft function, although whether this impacts long-term outcomes remains, at least in paediatrics, unclear [9, 13, 14]. This scepticism is partially reflected by the slower increase in the use of DCD organs for paediatric recipients in comparison to adult recipients. For example, in the USA, over the last 20 years there has been a 19x increase in adults receiving kidney transplants from DCD donors, whereas in paediatric recipients there has only been a 2.4x increase [1]. This could partially be explained by a lower proportion of paediatric donors being DCD donors than in adult donors (30% vs. 44% in 2024) [1], however, the majority of children still receive organs from adult donors and so the use of DCD organs for paediatric recipients should see a similar increase as in adults.

This study aims to compare the long-term outcomes of kidney transplants from DCD donors and DBD donors to paediatric recipients in the USA over a 26-year period.

## Materials and Methods

The Organ Procurement and Transplantation Network (OPTN) database is an online registry developed by United Network of Organ Sharing (UNOS) that contains all data pertaining to patient waiting lists, living and deceased organ donation, organ matching and organ transplants that have taken place in the U.S. since 1st October 1987 [15]. Data is added to the database at the point of listing a patient for transplant, at the point of donation and is updated at 6 months, 1-year and annually post-transplant with recipient outcome data.

OPTN registry data for all kidney transplants performed for recipients under the age of 18 years from deceased donors in the U.S. from October 1994 (date of first DCD transplant) until September 2020 were requested. Data collected included donor and recipient demographics, primary renal disease, number of prior transplants, dialysis status at transplantation, number of Human Leucocyte Antigen (HLA) mismatches, primary allograft non-function, delayed allograft function, allograft survival and patient survival time. All patients’ follow up data was based on their status on the registry in January 2021. Patients with no data recorded on the donor type were excluded. All variables collected and proportion of patients with missing data for each variable can be seen in Supplementary File 1.

All statistical analysis was carried out with IBM Statistical Package for Social Sciences (SPSS) Version 28 [16]. Propensity-Score-Matched (PSM) groups were created on a 1:2 basis based on recipient age, dialysis, prior-transplant status, year of transplantation, number of HLA mismatches and donor creatinine. Demographics were described for the whole cohort and the PSM cohort. Post-transplant outcomes including allograft and patient survival were compared between patients with transplants from DBD and DCD donors from the PSM group. Means and (95% confidence intervals) were reported to describe all numerical data, frequencies and percentages were used to describe categorical data. Independent T-test, chi-squared test and Analysis of Variance (ANOVA) was used for significance testing to compare groups of patients. Distribution analysis prior to group comparisons was carried out by visual assessment of histograms in order to identify the appropriate test to use. Patient and allograft survival at 1, 3, 5 and 10 years post-transplant by different eras of transplantation were estimated using Kaplan-Meier analysis and log-rank testing was used to assess comparisons. Multiple Multivariate Cox Regression Models for allograft and patient survival were also done from the PSM cohort in a sensitivity analysis. Bonferroni corrections were implemented to account for the number of comparisons made between the DBD and DCD group which left a threshold of significance of p < 0.01. Patients with missing data were excluded from proportional analysis for each analysis that required the variable in question.

The results from this data have been reviewed by all current members of the Paediatric Donation and Transplantation Working Group of the Ethical, Legal and Psychosocial Aspects of Organ Transplantation Section of the European Society of Organ Transplantation. The recommendations and suggestions that are included in this manuscript based on our results from this study were discussed, supported and agreed upon by all working group members prior to publication.

## Results

### Patients and Transplant Characteristics

Overall, during the study period there were 11,071 paediatric kidney transplants from deceased donors, of which 350 were from DCD donors.

Patients receiving allografts from DCD donors were significantly older than from DBD donors at 12.90 (12.45–13.37) years old vs. 11.52 (11.42–11.61) years old (p < 0.01). There was no significant difference in the distribution across different ethnicities (p = 0.36). The first DCD transplant in this cohort of patients took place in 1994 and rates of DCD transplantation have progressively increased since then. The mean follow up time for patients was 6.69 (6.59–6.79) years for DBD and 5.10 (4.69–5.52) years for DCD. Further details including underlying sex, underlying renal disease, dialysis status and number of prior transplants for both the baseline cohort and the PSM cohort can be seen in Table 1.

**TABLE 1:** Number and proportions of DBD and DCD transplants for different baseline characteristics for the overall cohorts and for the propensity score matched (PSM) cohorts. Patients with “unknown” listed as their primary renal disease were excluded from the proportions that were compared between DBD and DCD. Patients with no data recorded for each variable were excluded from analysis for their respective category.

Participant characteristics		DBD (n = 10,721)	DCD (n = 350)	p-value	DBD-PSM (n = 662)	DCD-PSM (n = 349)	p-value
Mean age (95% CI)		11.52 (11.42–11.61)	12.90 (12.45–13.37)	**<0.01**	12.84 (12.52–13.15)	12.90 (12.44–13.36)	0.41
Male (%)		6,231 (58.1)	194 (55.4)	0.31	366 (55.3)	193 (55.3)	0.99
Ethnicity (%)	White	4,475 (41.7)	149 (42.6)	0.75	255 (38.5)	149 (42.7)	0.19
	Black	2,624 (24.5)	87 (24.9)	0.87	170 (25.7)	86 (24.6)	0.71
	Hispanic	2,897 (27.0)	80 (22.9)	0.73	195 (29.5)	80 (22.9)	0.02
	Asian	388 (3.6)	18 (5.1)	0.13	23 (3.5)	18 (5.2)	0.19
	Other/Mixed	337 (3.1)	16 (4.6)	0.13	19 (2.9)	16 (4.6)	0.15
Underlying Renal Disease (% of known causes)	Unknown	1,304 (12.2)	36 (10.3)	0.29	66 (10)	35 (10)	0.98
	Cystic	669 (7.1)	20 (6.4)	0.61	39 (6.5)	20 (6.4)	0.91
	Pyelonephritis/Obstruction/Reflux	2,147 (22.8)	75 (23.9)	0.65	126 (21.1)	78 (23.9)	0.34
	Glomerulonephritis	3,135 (33.3)	112 (35.7)	0.37	234 (39.3)	112 (35.7)	0.3
	Hypertension/Vascular	444 (4.7)	17 (5.4)	0.56	29 (4.9)	17 (5.4)	0.71
	Hereditary/Metabolic	602 (6.4)	13 (4.1)	0.1	41 (6.9)	13 (4.1)	0.09
	Hypoplasia/Dysplasia	1,693 (18.0)	57 (18.2)	0.93	100 (16.8)	57 (18.2)	0.6
	Other	727 (7.7)	20 (6.4)	0.37	27 (4.5)	20 (6.4)	0.23
Era (%)	1990–1999	1933 (18.0)	11 (3.2)	**<0.01**	34 (5.1)	11 (3.2)	0.14
	2000–2009	4,030 (37.6)	106 (30.3)	**<0.01**	184 (27.8)	105 (30.1)	0.45
	2010–2020	4,758 (44.4)	233 (66.6)	**<0.01**	464 (67.1)	233 (66.8)	0.27
Number of Prior Transplants (%)	0	9,581 (89.4)	307 (87.7)	0.32	593 (89.6)	307 (88.0)	0.43
	1	1,040 (9.7)	41 (11.7)	0.21	65 (9.8)	40 (11.5)	0.41
	2	92 (0.9)	2 (0.6)	0.56	3 (0.5)	2 (0.6)	0.79
	3	8 (0.1)	0 (0)	-	1 (0.2)	0 (0)	-
Favourable Number of HLA Mismatches (%)		2050 (19.1)	60 (17.1)	0.34	122 (18.4)	60 (17.2)	0.62
Pre-emptive Transplant (%)		2,233 (20.8)	66 (18.9)	0.37	122 (18.4)	66 (18.9)	0.85
Mean Follow Up Time in Years (95% CI)		6.69 (6.59–6.79)	5.10 (4.69–5.52)	**<0.01**	5.02 (4.72–5.32)	5.12 (4.68–5.55)	0.37
Mean Cold Ischaemia Time in Hours (95% CI)		14.59 (14.44–14.74)	15.27 (14.61–15.94)	0.06	13.96 (13.36–14.56)	15.27 (14.61–15.94)	**<0.01**
Mean Warm Ischaemia Time in Minutes (95% CI)		-	12.01 (10.73–13.30)	-	-	16.36 (15.15–17.58)	-
Mean Donor Creatinine in mg/DL (95% CI)		0.96 (0.94–0.97)	0.81 (0.78–0.85)	**<0.01**	0.83 (0.80–0.85)	0.81 (0.78–0.85)	0.23

Bold values show statistical significance of p < 0.01.

Patients with transplants from DCD donors were more likely to have transplants with an unfavourable number of HLA mismatches (4, 5 or 6 mismatches) with 82.9% of transplants having an unfavourable HLA mismatch compared to 80.7% of DBD donors, although this was not statistically significant (p = 0.34). There was no significant difference in the mean cold ischaemia time between DCD and DBD donors – 15.27 h (14.61–15.94 h) vs. 14.59 h (14.44–14.74 h), p = 0.06. The mean warm ischaemia time (WIT) for all DCD transplants was 12.01 min (10.7–13.3).

### Post-Transplant Outcomes

Patients with transplants from DCD donors were significantly more likely to have delayed graft function (DGF) than DBD donors (n = 71, 20.3% vs. n = 60, 9.1%, p < 0.01) however there was no significant difference in the rates of primary non-function (PNF) between the donor types (n = 4, 1.2% vs. n = 5, 0.8%, p = 0.52). There was also no significant difference in the incidence of renal vessel thrombus (n = 9, 2.6% vs. n = 11, 1.7%, p = 0.31).

The mean creatinine (mg/dL) at the point of hospital discharge post-transplant was significantly higher in patients with transplants from DCD donors – 2.21 mg/dL (1.99–2.43 mg/dL) than in patients with transplants from DBD donors - 1.36 mg/dL (1.24–1.48 mg/dL) (p < 0.01).

**FIGURE 1:**
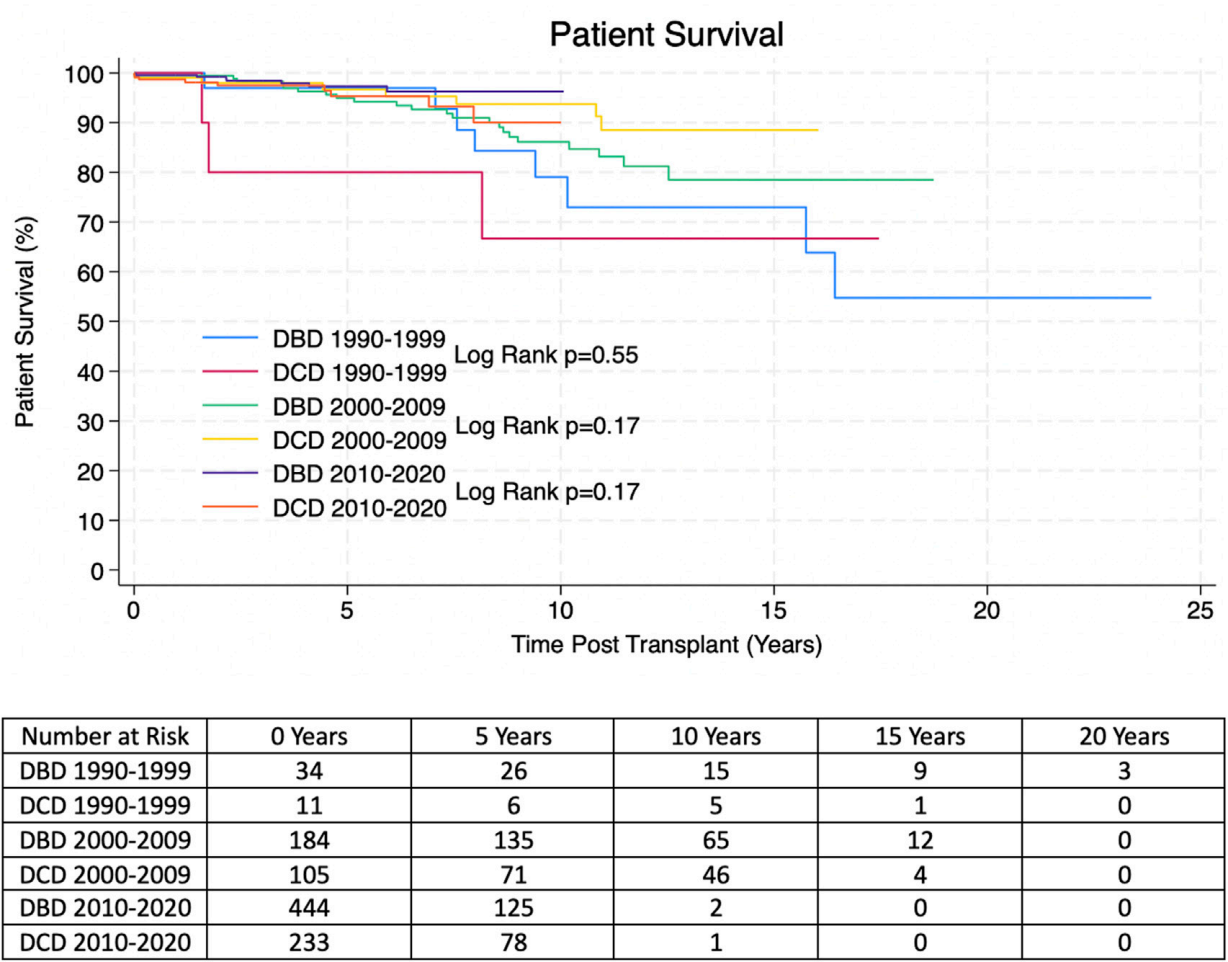
Estimated Kaplan-Meier allograft survival post-transplant for transplants from DBD and DCD donors from the propensity score matched (PSM) cohort. P-values were derived from the Log Rank Test.

**FIGURE 2:**
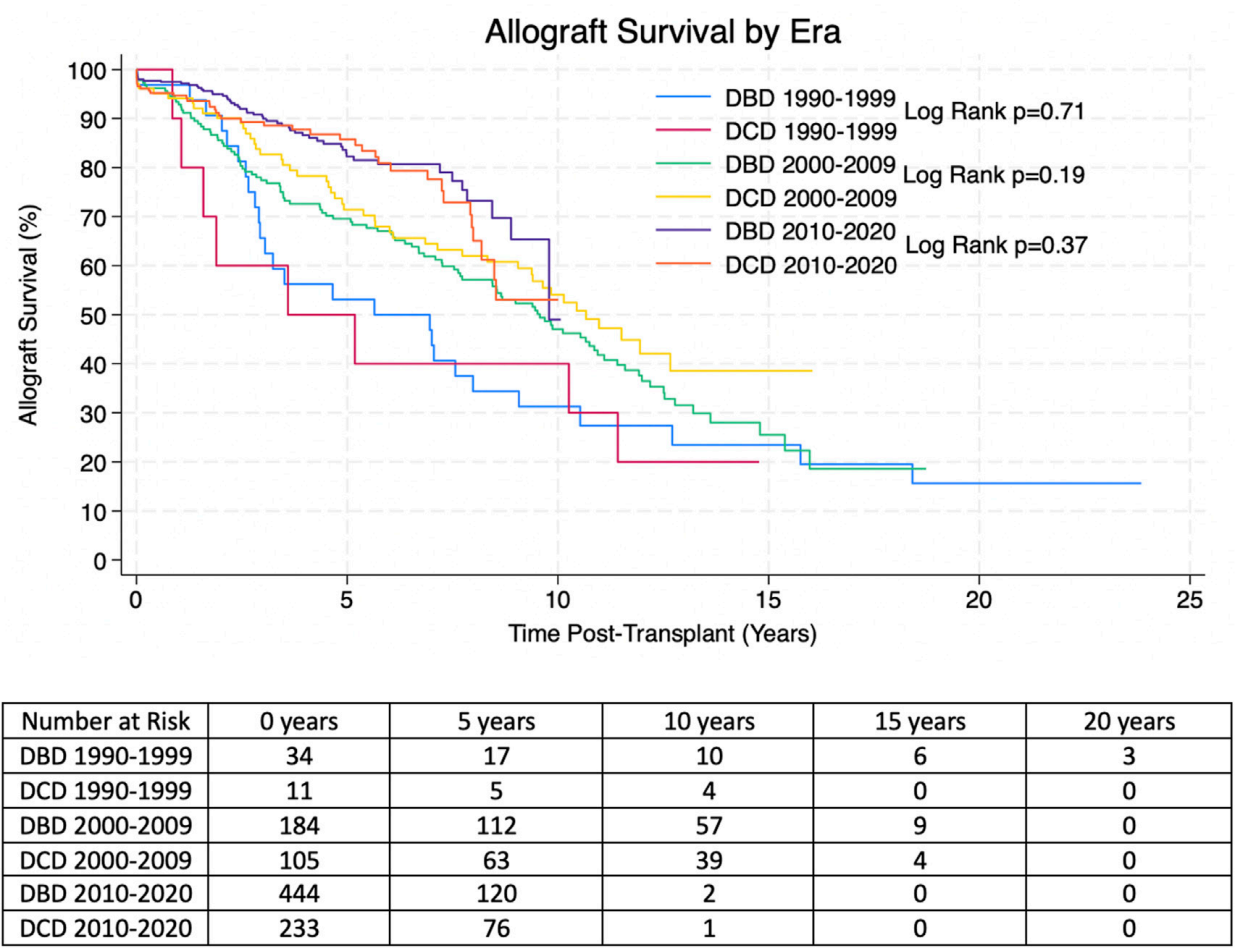
Estimated Kaplan-Meier patient survival post-transplant for transplants from DBD and DCD donors from the propensity score matched (PSM) cohorts. P-values were derived from the Log Rank Test.

Kaplan-Meier allograft and patient survival was stratified into three categories representing three different eras of transplantation–Pre-2000, 2000–2009 and 2010-present. Within each time period there was no significant difference in allograft or patient survival between transplants from DBD and DCD donors. 1, 3, 5 and 10 years allograft and patient survival for these patients is summarized in Tables 2, 3 and survival curves can be seen in Figures 1, 2.

**TABLE 2:** Overall estimated Kaplan-Meier allograft survival for transplants from DBD and DCD donors in the propensity score matched (PSM) cohort at 1, 3, 5 and 10 years post-transplant. P-values were derived with the Log Rank Test.

Patient group	1 year	3 years	5 years	10 years	p-value
DBD 1990–2000	93.8%	62.5%	50.0%	31.0%	p = 0.71
Number at risk	*31*	*21*	*17*	*10*	
DCD 1990–2000	80.0%	50.0%			
Number at risk	*9*	*6*			
DBD 2000–1,010	92.9%	76.8%	70.0%	47.1%	p = 0.19
Number at risk	*169*	*130*	*112*	*57*	
DCD 2000–2010	94.1%	82.7%	70.2%	54.1%	
Number at risk	*95*	*77*	*62*	*38*	
DBD 2010–2020	97.5%	90.4%	82.2%	49.0%	p = 0.37
Number at risk	*357*	*209*	*120*	*2*	
DCD 2010–2020	94.7%	89.3%	85.8%	53.1%	
Number at risk	*182*	*124*	*76*	*1*	

Italic values represent number at risk at each time point.

**TABLE 3:** Overall estimated Kaplan-Meier patient survival for transplants from DBD and DCD donors from the propensity score matched (PSM) cohort at 1, 3, 5 and 10 years post-transplant. P-values were derived with the Log Rank Test.

Patient group	1 year	3 years	5 years	10 years	p value
DBD 1990–2000	97.0%	97.0%	97.0%	79.0%	p = 0.55
Number at risk	*33*	*31*	*26*	*15*	
DCD 1990–2000	90.0%	80.0%	66.7%	66.7%	
Number at risk	*10*	*7*	*6*	*5*	
DBD 2000–1,010	99.5%	98.2%	95.0%	86.1%	p = 0.17
Number at risk	*179*	*156*	*135*	*65*	
DCD 2000–2010	99.0%	98.0%	96.7%	93.8%	
Number at risk	*97*	*84*	*71*	*46*	
DBD 2010–2020	99.5%	98.5%	97.3%	96.3%	p = 0.17
Number at risk	*359*	*211*	*125*	*2*	
DCD 2010–2020	98.7%	97.5%	95.3%	90.0%	
Number at risk	*187*	*129*	*78*	*1*	

Italic values represent number at risk at each time point.

After PSM, within the new cohort the following variables were equally matched: dialysis status at transplantation, year of transplant, number of HLA mismatches, donor creatinine and recipient age. Multivariate Cox Regression analysis was undertaken with multiple different models accounting for other potential residual confounders including some that were stratified by era of transplantation, early graft failure, early death and follow up time for allograft and patient survival as a sensitivity analysis. This found that DBD vs. DCD transplantation consistently had no statistically significant impact on allograft and patient survival. Hazard ratios and significance for the different models can be seen for these in Tables 4, 5.

**TABLE 4:** Sensitivity analysis with Multivariate cox regression models for allograft survival in the propensity score matched (PSM) cohorts.

Variables	Hazard ratio	p-value	95% CI
Model 1			
DCD	0.93	0.56	0.72–1.19
Model 2			
DCD	0.9	0.44	0.70–1.17
CIT	0.1	0.11	0.99–1.02
Ethnicity	0.93	0.06	0.86–1.00
Model 3			
DCD	0.93	0.57	0.73–1.19
Number of Previous Transplants	1	0.95	0.74–1.39
Model 4 - Stratified by Era of Transplantation			
DCD	0.95	0.69	0.74–1.22
Model 5 - Stratified by Early Graft Failure (<1 year post-transplant)			
DCD	1.57	0.15	0.85–2.87
Model 6 - Stratified by Late Graft Failure (>1 year post-transplant)			
DCD	0.85	0.25	0.65–1.12
Model 7 - Stratified by Short Follow Up Time (<1 year post-transplant)			
DCD	1.92	0.1	0.88–4.21
Model 8 - Stratified by Long Follow Up Time (>1 year post-transplant			
DCD	0.85	0.24	0.66–1.11
Model 9 - Stratified by Very Long Follow Up Time (>5 years post-transplant)			
DCD	0.88	0.47	0.62–1.25

**TABLE 5:** Sensitivity analysis with Multivariate cox regression models for patient survival in the propensity score matched (PSM) cohorts.

Variables	Hazard ratio	p-value	95% CI
Model 1			
DCD	0.94	0.83	0.54–1.63
Model 2			
DCD	0.95	0.87	0.54–1.69
CIT	1.02	0.09	0.99–1.05
Ethnicity	0.96	0.60	0.81–1.13
Model 3			
DCD	0.96	0.88	0.55–1.67
Number of Previous Transplants	1.41	0.25	0.79–2.55
Model 4 - Stratified by Era of Transplantation			
DCD	0.99	0.98	0.57–1.73
Model 5 - Stratified by Early Death (<1 year post-transplant)			
DCD	0.94	0.83	0.54–1.64
Model 6 - Stratified by Late Death (>1 year post-transplant)			
DCD	2.2	0.3	0.50–9.91
Model 7 - Stratified by Short Follow Up Time (<1 year post-transplant)			
DCD	2.77	0.16	0.68–11.3
Model 8 - Stratified by Long Follow Up Time (>1 year post-transplant			
DCD	0.71	0.29	0.37–1.35
Model 9 - Stratified by Very Long Follow Up Time (>5 years post-transplant)			
DCD	0.75	0.63	0.23–2.42

## Discussion

This observational retrospective study describes the clinical outcomes of the largest cohort of paediatric kidney transplant recipients from DCD donors in the literature. Overall, we have shown that kidney transplants from DCD donors have equivocal outcomes to kidney transplants from DBD donors in paediatric recipients.

One of the positive trends identified in this data is that there does not seem to be any racial disparities within DCD transplantation which unfortunately does still exist in access to transplantation as a whole [17].

One of the main concerns with allografts from DCD donors is the impact of the WIT which is associated with these allografts and the higher rates of DGF. Our mean WIT was 12.01 min which is better than or equivocal to other studies [18, 19]. The higher risk of DGF with DCD allografts has been widely reported in the literature and is also found in our data and is also reflected by the higher creatinine levels at discharge in these patients [10, 18–20]. Currently, we are unclear as to what potential downstream effects this higher creatinine level might have, while it does not appear to impact allograft survival, we do not have data to clarify if it affects other aspects such as CKD progression. However, our rate of DGF in DCD allografts was not quite as high as reported in adults [18]. DGF occurs due to a multitude of reasons including but not limited to increased cold ischaemia time, increased WIT, donor hypertension and obesity and pre-transplant dialysis [21–23]. The literature on adult patients suggests that DGF can be associated with poorer long-term outcomes including shorter death-censored allograft survival and higher rates of acute rejection and so it is important to try and avoid this [10, 18, 21, 23]. However, one adult study found that DCD allografts may be more resilient, and that DGF in a DCD allograft has less impact on long-term outcomes than DGF in DBD allografts [10]. Nevertheless, strategies to try and reduce the incidence of DGF remain important. Some adult studies have found that the use of both hypo- and normothermic machine perfusion of the allografts prior to transplantation can significantly reduce the incidence of DGF and positively impact long-term allograft survival when compared to static cold storage [24–28]. Higher rates of DGF in DCD allografts may prompt retrieval teams to consider the use of these perfusion techniques more often when retrieving DCD allografts to improve their outcomes. In addition to perfusion techniques, studies have found that reducing CIT can also help mitigate the increased risk of DGF in DCD kidneys [9].

Reassuringly, despite higher rates of DGF, the long-term outcomes of transplants from DCD donors appear equivocal to those from DBD donors with regards to allograft and patient survival. This is similar to some of the adult and paediatric evidence in the literature [11, 18–20], although some studies have not found this to be the case and have shown increased rates of late-allograft loss [12]. Some of the reasons our study may vary from that is our larger sample size and longer follow up time; that study referred to late-allograft loss as 4 years-post transplant, however they only had 4 years follow up data for 31 DCD patients, whereas we have 4 years follow up data for 183 DCD patients [12]. The warm ischaemia time in our cohort was also lower than some other studies; evidence shows that warm ischaemia time correlates with poorer outcomes and so this may also account for our positive [14]. Our results show DCD donors are a valuable resource that provide good outcomes, comparable to those of DBD donors, and have the potential to facilitate more kidney transplants for paediatric recipients. While some centres may be hesitant to use these kidneys for paediatric recipients, we have now shown that they can have good clinical outcomes, in line with DBD kidneys and so one should consider utilizing these as a viable transplant option. Furthermore, one adult study has shown that there is a significant survival benefit of accepting a DCD organ offer compared to remaining on the waiting list for an allograft from a DBD donor [29]. Utilizing DCD donors for paediatric transplant recipients can also lead to an increase in pre-emptive transplantation which is known to be associated with better clinical outcomes [30].

In countries that do not perform DCD in children any approach to consider it will need to address the specific national reasons. If elective withdrawal of life-sustaining therapies at end-of- life in ICU is not the national norm, then DCD can only follow a much more involved discussion about usual end-of-life practices in ICU which may need legal changes/clarification, as well as changes in medical norms. If DCD is already practiced in adults, though not in children, this is a far easier argument. The clear global evidence for safe paediatric DCD means that with appropriate training and infrastructure, maintaining the distinction is unethical, as it prevents children, and their parents, from donating organs when they die, leading to the deaths of other children based on indefensible age discrimination. Initial controlled DCD will, of course, be easier to introduce than donation following failed acute resuscitation, which many countries with normalized paediatric DCD processes do not practice.

The main strength of this study is its large sample size, to date, the largest reported in the paediatric population, which allows significant trends to be identified reliably. It also presents a long follow-up time further distinguishing it from previous studies. However, the study could be limited by the potential heterogeneity of the data. While we have tried to control for some confounders by using propensity score matching, Multivariate Cox Regression models and sensitivity analysis; variables such as the use of machine perfusion, immunosuppression agents, centre effect, rejection episodes, CKD progression, donor age and organ quality were not reported and not accounted for in analysis and could be significant confounders. Furthermore, this is a retrospective registry study, which means some data may not be that reliable, many variables are unable to be accounted for and patients risk being lost to follow up. Our data is also based on the American allocations system and transplant practice which could limit the generalizability to other countries, however our positive results should remain encouraging to clinicians worldwide. Additionally, the allocation system in the USA since 2014, which allocates kidneys with a higher expected allograft survival to patients with the highest potential life-expectancy, may also be a confounding factor. This is because kidneys from DCD donors are currently considered to have a risk of lower expected allograft survival than DBD kidneys and so are not allocated in the same way [31]. We have tried to control for this by propensity score matching our group so that baseline characteristics between recipients in the DBD and DCD group do not differ, however some confounding may remain. Our current data does not support the notion that DCD kidneys are inferior organs, however further and more in depth research is required to confirm this and therefore allocation policies and clinicians’ views may need to adjust to reflect this as more evidence becomes available.

Further studies are needed to explore the outcomes of DCD transplants around the world, and to look into other outcome data such as rejection rates. It would also be helpful to further investigate any potential factors that can reduce the rate of DGF in paediatric DCD transplant recipients. Qualitative and sociological studies are also important to aid understanding of the public and political views on DCD practices in countries that so far do not permit it to better understand the barriers facing DCD transplant programmes.

## Conclusion

In conclusion, within the limits of a retrospective observational study, paediatric patients receiving a kidney transplant from a DCD donor have the same long-term outcomes as those receiving transplants from DBD donors, despite a higher rate of DGF. Innovation in perfusion techniques may help reduce the rate of DGF in DCD kidneys. However, there may still be some potential residual confounding in our data despite using propensity-score matched data and cox regression models to control for these. While keeping this in mind, we recommend that allografts from DCD donors should be utilized more, and considered a viable option for paediatric recipients, as they can have excellent outcomes and offer the potential opportunity of reducing waitlist times and facilitating more transplants potentially leading to improved quality of life in these children. Allocation systems may need to be updated to reflect these findings. On behalf of the ELPAT (Ethical, Legal and Psychosocial Aspects of Transplantation) section at the European Society for Organ Transplantation, given the excellent results presented in this manuscript we suggest that findings should inform clinical decision making and policy discussions, particularly in countries where DCD is not yet allowed but is being considered. As groups attempt to unify the neurological determination of death criteria globally [32], we consider that consistent global approaches to the circulatory determination of death and DCD are legally and ethically justified.

## Data Availability

The data analyzed in this study is subject to the following licenses/restrictions: All clinical data is available as requested from the OPTN database. Requests to access these datasets should be directed to https://optn.transplant.hrsa.gov/data/view-data-reports/request-data/.

## Abbreviations

ANOVA, Analysis of Variance; DGF, Delayed Graft Function; DBD, Donation after brainstem determination of death; DCD, Donation after circulatory determination of death; HLA, Human Leucocyte Antigen; OPTN, Organ Procurement and Transplantation Network; PNF, Primary Non-Function; PSM, Propensity Score Matched; SPSS, Statistical Package for Social Sciences; UNOS, United Network of Organ Sharing; WIT, Warm Ischaemia Time.
