# Supplementary material for: Long-Term Outcomes of Pediatric Kidney Transplants From DCD and DBD Donors: A Comparative OPTN Study
Source: Transpl Int. 2025 Oct 9;38:14706. doi: 10.3389/ti.2025.14706 (PMC12548547; doi:10.3389/ti.2025.14706)
Supplement: Supplementary file 1 [file Table1.docx]

*Supplementary File 1: Table showing all variables collected and proportion of missing variables for both DBD and DCD transplants.*

| Variable | Number of DBD patients with missing data | Proportion of DBD patients with complete data (%) | Number of DCD patients with missing data | Proportion of DCD patients with complete data (%) |
| --- | --- | --- | --- | --- |
| Patient Registration Number | 0 | 100% | 0 | 100% |
| Previous Transplant | 0 | 100% | 0 | 100% |
| Required Re-Transplantation | 0 | 100% | 0 | 100% |
| Dialysis Status at Transplant | 483 | 96.2% | 5 | 98.6% |
| Recipient ABO | 0 | 100% | 0 | 100% |
| Renal Disease | 0 | 100% | 0 | 100% |
| Recipient Ethnicity | 0 | 100% | 0 | 100% |
| Recipient Sex | 0 | 100% | 0 | 100% |
| Transplant Date | 0 | 100% | 0 | 100% |
| Delayed Allograft Function | 131 | 98.9% | 4 | 98.9% |
| Creatinine at Discharge | 546 | 95.7% | 7 | 98.0% |
| Left or Right Kidney | 0 | 100% | 0 | 100% |
| En-bloc Kidney | 0 | 100% | 0 | 100% |
| Simultaneous Transplant | 0 | 100% | 0 | 100% |
| Other Organs | 0 | 100% | 0 | 100% |
| HLA Mismatch | 1 | 99.9% | 0 | 100% |
| HLA Mismatch Level | 60 | 99.5% | 0 | 100% |
| Donor ABO | 0 | 100% | 0 | 100% |
| Donor Type | 0 | 100% | 0 | 100% |
| Warm Ischaemia Time | n/a | n/a | 135 | 61.9% |
| Donor Creatinine | 2088 | 83.7% | 1 | 99.7% |
| ABO Incompatible | 0 | 100% | 0 | 100% |
| Recipient Age | 0 | 100% | 0 | 100% |
| Cold Ischaemia Time | 961 | 92.4% | 24 | 93.2% |
| Primary Non Function | 0 | 100% | 0 | 100% |
| Graft Thrombus | 0 | 100% | 0 | 100% |
| Death with a Functioning Graft | 0 | 100% | 0 | 100% |
| Graft Failure | 1 | 99.9% | 0 | 100% |
| Graft Survival | 67 | 99.5% | 4 | 98.9% |
| Graft Failure Death Censored | 0 | 100% | 0 | 100% |
| Patient Status | 64 | 99.5% | 4 | 98.9% |
| Patient Status Date | 1 | 99.9% | 4 | 98.9% |
| Death | 1 | 99.9% | 0 | 100% |
| Patient Survival | 68 | 99.5% | 4 | 98.9% |
